# Altered Precipitation Impacts on Above- and Below-Ground Grassland Invertebrates: Summer Drought Leads to Outbreaks in Spring

**DOI:** 10.3389/fpls.2016.01468

**Published:** 2016-10-06

**Authors:** Marcel D. Torode, Kirk L. Barnett, Sarah L. Facey, Uffe N. Nielsen, Sally A. Power, Scott N. Johnson

**Affiliations:** ^1^School of Biosciences, Cardiff UniversityCardiff, UK; ^2^Hawkesbury Institute for the Environment, Western Sydney University, RichmondNSW, Australia

**Keywords:** aboveground–belowground interactions, arthropods, climate change, multi–trophic interactions, rainfall extremes, root herbivores, soils

## Abstract

Climate change is predicted to result in altered precipitation patterns, which may reshape many grassland ecosystems. Rainfall is expected to change in a number of different ways, ranging from periods of prolonged drought to extreme precipitation events, yet there are few community wide studies to accurately simulate future changes. We aimed to test how above- and below-ground grassland invertebrate populations were affected by contrasting future rainfall scenarios. We subjected a grassland community to potential future rainfall scenarios including ambient, increased amount (+50% of ambient), reduced amount (–50% of ambient), reduced frequency (no water for 21 days, followed by the total ambient rainfall applied in a single application) and summer drought (no rainfall for 13 weeks during the growing season). During Austral spring (September 2015), we sampled aboveground invertebrates, belowground macro invertebrates and nematodes. Aboveground communities showed a significant response to altered rainfall regime with the greatest effects observed in summer drought plots. This was mostly due to a large increase in sucking herbivores (658% higher than ambient plots). Plots experiencing summer droughts also had higher populations of parasitoids, chewing herbivores and detritivores. These plots had 92% more plant biomass suggesting that primary productivity increased rapidly following the end of the summer drought 5 months earlier. We interpret these results as supporting the plant vigor hypothesis (i.e., that rapid plant growth is beneficial to aboveground invertebrates). While belowground invertebrates were less responsive to altered precipitation, we observed a number of correlations between the abundances of above- and below-ground invertebrate groups under ambient rainfall that dissipated under altered rainfall regimes. Mechanisms underpinning these associations, and reasons for them to become decoupled under altered precipitation regimes (we term this ‘climatic decoupling’), remain speculative, but they provide the basis for formulating hypotheses and future work. In conclusion, we predict that shifts in rainfall patterns, especially summer drought, will likely have large, but probably short-term, impacts on grassland invertebrate communities. In particular, sucking herbivores show sensitivity to precipitation changes, which have the potential to cascade through the food chain and affect higher trophic levels.

## Introduction

Invertebrates make up 96% of known terrestrial species, with an estimated contribution to ecosystem services worth $60 billion per year in the US alone (Losey and Vaughan, 2006). Invertebrates are the main faunal component of grassland ecosystems and key to ecosystem functioning, contributing to services such as soil formation, pollination and population control (Curry, 1994; Weisser and Siemann, 2004). Grasslands are globally important ecosystems, underpinning livestock production and regulating our climate (Gibson, 2009), with carbon sequestration by grasslands estimated to be worth over $200 per hectare (Daily, 1997).

Climate change models predict altered precipitation patterns and an increased number of extreme precipitation events (IPCC, 2014), which will likely impact grasslands and the invertebrates within them. Changes in rainfall could be highly variable. For example, Australian rainfall records have shown recent increases in wet and dry extremes as well as greater seasonal variation, thought to be partly explained by climate change (Garnaut, 2011). Altered rainfall patterns can have direct effects on invertebrates, such as heavy rainfall events causing physical damage during flight, to reducing foraging efficiency and increasing migration times (Barnett and Facey, 2016). On the other hand, droughts can cause desiccation of above- and below-ground invertebrates, often decreasing the viability and survival of the eggs and larvae (e.g., Johnson et al., 2010; Gantz and Lee, 2015). Predicting the impacts of changes in rainfall on invertebrate communities is complicated by the fact that altered rainfall is unlikely to affect all invertebrates in the same way. For example, the impact of drought on soft bodied invertebrates, such as isopods and myriapods, is likely to be greater than the effects on arachnids and insects which have a waxy cuticle that serves to reduce water loss (Berridge, 2012).

Indirect effects of water stress, such as those mediated by plants, can also affect invertebrates differently (Koricheva et al., 1998). Sucking herbivores, for example, may benefit from an increased concentration of nitrogenous compounds in plant foliage following water stress, but only when plants enter a recovery phase (i.e., turgor pressure returns). Huberty and Denno (2004) termed this the ‘pulsed-stress hypothesis,’ but also noted that increased levels of plant defensive compounds following water stress often reduce the abundance of chewing herbivores. In contrast, the plant vigor hypothesis has been proposed based on observations that some phytophagous insects choose vigorous, fast-growing parts of plants to feed on (Price, 1991). Fast growing plant tissue is thought to have higher nutrient availability, greater osmotic potential and be relatively low in fiber and lignified tissue (Price, 1991, 2003). Decreased rainfall or altered patterns of rainfall, causing plant dieback, might subsequently stimulate such vigorous re-growth of some plant species when precipitation patterns return to ambient levels.

The group or guild specific responses of invertebrates to altered precipitation patterns are likely to alter the interactions occurring between species, and subsequently the structure of communities (Voigt et al., 2003). Yet, relatively few studies explore the impact of climate change at the community level, with still fewer including both above- and below-ground invertebrates (McKenzie et al., 2013). Altered rainfall can have variable effects on the interactions between invertebrates. For example, Staley et al. (2007) showed that a root chewer can negatively affect the performance of an aboveground leaf miner, but this relationship breaks down under drought conditions. In contrast, some interactions only become apparent under drought conditions; belowground herbivores have been shown to have positive effects on leaf mining flies under reduced rainfall compared with ambient conditions (Staley et al., 2008). The nature of the water stress can also differentially impact above-belowground interactions; Tariq et al. (2013) showed that under a moderate drought stress, root herbivory increased plant chemical defenses, reducing the performance and abundance of a specialist herbivore. In highly drought stressed plants, however, this response did not occur.

Future precipitation regimes may therefore have a range of impacts on above- and below-ground invertebrates, and potentially modify linkages and interactions between these two groups. Experiments often use simplified pot and lab experiments, which are useful for teasing apart causal relationships but cannot fully incorporate synergies between the direct and indirect effects of altered precipitation across multiple trophic levels. Above- and below-ground linkages observed in such experiments, for instance, do not necessarily represent what happens in field situations (e.g., Vandegehuchte et al., 2010; but see Johnson et al., 2013). Community-level field experiments that incorporate background climatic variation and simulate a range of precipitation regimes, looking beyond just reduced and increased rainfall scenarios (e.g., seasonal and frequency related changes), could prove provide a more realistic simulation of such climatic change (Jentsch et al., 2007). Nonetheless, measuring changes in both above- and below-ground communities is destructive and imposes legacy effects so these experiments provide a ‘snapshot’ of above- and below-ground community changes rather than detailed temporal information.

We aimed to test how above- and below-ground grassland invertebrate populations were affected by contrasting future rainfall scenarios. To achieve this, we used a unique field-based community experiment in southeast Australia that applied ambient levels of precipitation together with four predicted precipitation patterns in a grassland ecosystem. We identified the effects of altered rainfall patterns on the abundance of invertebrate taxonomic groups, feeding guilds and the structure of the community as a whole. Additionally, we explored potential associations between above- and below-ground invertebrate communities and tested whether these were affected by altered precipitation patterns.

## Materials and Methods

### Experimental Site and Shelters

We used the DRI-Grass (Drought and Root herbivore Impacts on a Grassland ecosystem) experimental platform for this research. This platform applies different rainfall regimes in a grassland ecosystem based in Richmond, New South Wales at the Western Sydney University Hawkesbury campus (33°36′40″ S, 150°44′26.5″ E). The DRI-Grass experiment consists of 48 permanent rain exclusion shelters (1.8 m × 2.0 m area; i.e., 3.6 m × 3.6 m) constructed from stainless steel frames and clear acrylic Perspex roofs. These shelters allow five rainfall scenarios to be simulated comprising 12 plots with (i) ambient (water applied in “real time” immediately after rainfall events), (ii) reduced amount (-50% of ambient), (iii) reduced frequency (no water for 21 days, followed by the total ambient rainfall applied in a single application) as well as six plots with, (iv) increased amount (+50% of ambient), and (v) summer drought (no rainfall for 13 weeks in the summer, 17 December to 27 March). Six plots of each of the 12 ambient, reduced amount and reduced frequency plots had been inoculated with scarab larvae as part of a concurrent, but separate, experiment which we accounted for as a covariate factor in our analysis (see Statistical analysis below). To assess differences between watering regimes, soil moisture readings are automatically taken every 15 min from TDR probes, and a daily mean was calculated (see Power et al., 2016 for full details of the DRI-Grass platform).

The grassland community beneath each shelter typically consisted of *Axonopus fissifolius* (C_4_ grass), *Cymbopogon refractus* (C_4_ grass), *Eragrostis curvula* (C_4_ grass), *Hypochaeris radicata* (forb), *Microlaena stipoides* (C_3_ grass), *Paspalum dilatatum* (C_4_ grass), *Plantago lanceolata* (forb) and *Setaria parviflora* (C_4_ grass). The soil is characterized as a sandy loam of moderate to low fertility, with a low organic matter content and low water holding capacity (see Barton et al., 2010 for full details). DRI-Grass was constructed in May 2013 and irrigation regimes commenced in June 2013. Aboveground plant material is harvested at the end of both the cool (i.e., October) and growing season (i.e., April) and plant biomass is estimated for each plot.

### Invertebrate Collection

Three sampling methods were used to collect aboveground invertebrates, belowground macro-invertebrates and nematodes during 11–21 September 2015, prior to harvesting aboveground plant material in the following week. Vacuum (‘G-vac’ device) sampling, a proven quantitative technique (Brook et al., 2008), was used to capture aboveground invertebrates. The ‘G-Vac’ sampler was an adapted petrol-powered device (SH 86C, Sithl AG & Co. KG. Germany) fitted with an organza bag to catch invertebrates. This was passed under each rain exclusion shelter for 20 s in a zigzag pattern on full throttle. Samples were placed in zip lock bags and frozen until identification in the laboratory under a dissecting microscope (SZ51, Olympus, Japan). Belowground macro invertebrates were sampled by excavating two 25 cm × 10 cm × 20 cm (length × width × depth) trenches under each rain exclusion shelter. Macro invertebrates were stored into ethanol and frozen until identification in the laboratory. Both above- and below-ground samples were identified to at least order level, other than two groups which were identified to sub-class only (Acari and Collembola). A number of groups were also identified to sub order or family level, for more accurate identification of feeding guilds (see **Supplementary Tables 1** and **2** for the groups identified and guild classifications).

Two additional soil cores (3.5 cm diameter, 10 cm depth), one from each side of the plot, were collected for extraction of nematodes, at the same time as sampling belowground macro fauna. These were combined per plot to form a composite sample, gently homogenized and subsampled, with nematodes extracted from 50 g soil (wet weight) using a modified Baermann funnel technique (Baermann, 1917), over 3 days. The nematodes were then counted, assigned to feeding groups using morphological characteristics, and the numbers converted to individuals per kg dry soil. Nematode samples were collected as part of ongoing monitoring for interactive responses to herbivore addition and rainfall manipulations (i.e. Ambient, Reduced amount, and Reduced frequency treatment plots only), but were used in this study to investigate above- and below-ground linkages further.

### Statistical Analyses

**FIGURE 1:**
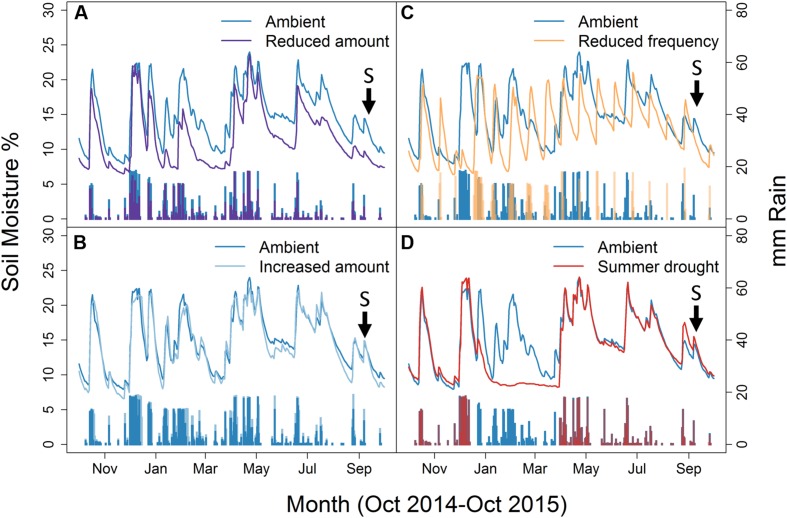
**Soil moisture and mm rain applied to ambient plots relative to; **(A)** Reduced amount, **(B)** Increased amount, **(C)** Reduced frequency, and **(D)** Summer drought.** Lines show the Soil moisture, while the bars represent mm of rain applied. Arrows and ‘S’ indicates the time sampling took place. Weekly data shown from October 2014 to October 2015.

Statistical analyses were undertaken in R, version 3.2.2 (R Core Team, 2016). To confirm the effect of the applied watering treatment on soil moisture, we tested the effects of the applied rainfall regimes on soil moisture data from October 2014 to October 2015, using a repeated measures linear mixed effects model [package nlme, lme(); Pinheiro et al., 2016]. Repeated measures were used because of the recurring nature of soil moisture measurements, which were recorded as a proportion (%) and therefore values were transformed prior to analysis using the logit() function (package *car*; Warton and Hui, 2011). The model used rainfall treatment and month as interactive fixed effects, with month nested within plot which was included as a random (mixed) effect. Interactive effects were tested first, followed by individual effects (Crawley, 2013). Custom *post hoc* comparisons were performed based on visual inspection (**Figure 1**) for significant factors [glht(), *multcomp*; Hothorn et al., 2008].

The effect of rainfall treatment on plant biomass from the October harvest was evaluated with a linear model using a Chi Square test, followed by a Tukey test.

Separate analyses were undertaken for each of the three different sampling methods (aboveground invertebrate vacuum sampling, belowground macro fauna and soil nematodes). Groups of invertebrates (separated by Order or feeding guild identity) that were captured infrequently - i.e., found in <10% of samples or constituting <50 individuals in total - were excluded to permit statistical analysis (*sensu* Hillstrom and Lindroth, 2008).The effects of altered rainfall on invertebrate abundance were tested using generalized linear models (GLMs). Models contained a negative binomial error structure to account for overdispersion of the data [glm.nb(), *MASS*; Venables and Ripley, 2003] in all but three groups for which model dispersion parameters <1.7; these were analyzed using Poisson error structures. In order to determine the significance of the rainfall treatment, the full model described above was compared to a reduced model without rainfall treatment as a factor (likelihood ratio test). A concurrent experiment inoculated half of the ambient, reduced frequency and reduced amount plots with scarabs; therefore herbivore treatment was included in models as a covariate to account for this. Additionally, due to the documented effect of vegetation complexity on suction sampling efficiency, plant biomass was also included as a covariate when analyzing the aboveground abundance data (Brook et al., 2008; Facey and Torode, 2016). The model fit was determined by inspecting residual plots.

The effect of altered rainfall regimes on the community composition of invertebrates was analyzed using permutational multivariate analysis of variance [PERMANOVA, adonis() in the *vegan* package; Oksanen et al., 2016], with rainfall treatment included as a fixed effect.

A Pearson’s correlation matrix was used to explore linkages between above- and below-ground invertebrate groups for each rainfall treatment separately. In order to avoid type II errors, only highly statistically significant (*P* < 0.01) results from the matrix were investigated further. A mixed model was used to show which of the highly significant correlations appeared to have a linear relationship; those that did not appear to have a linear relationship are not shown. Models used the abundance of two invertebrate groups that were significantly correlated, with the abundance of the aboveground group explained as a function of the abundance of the belowground group, with plot as a random effect. The fit of the model was assessed using residual plots and significance was determined using an ANOVA. Plots treated with additional root herbivory may have altered the relationships between above-below-ground invertebrates, so were not included in the analysis of above-belowground associations.

## Results

### Soil Moisture and Plant Responses

**FIGURE 2:**
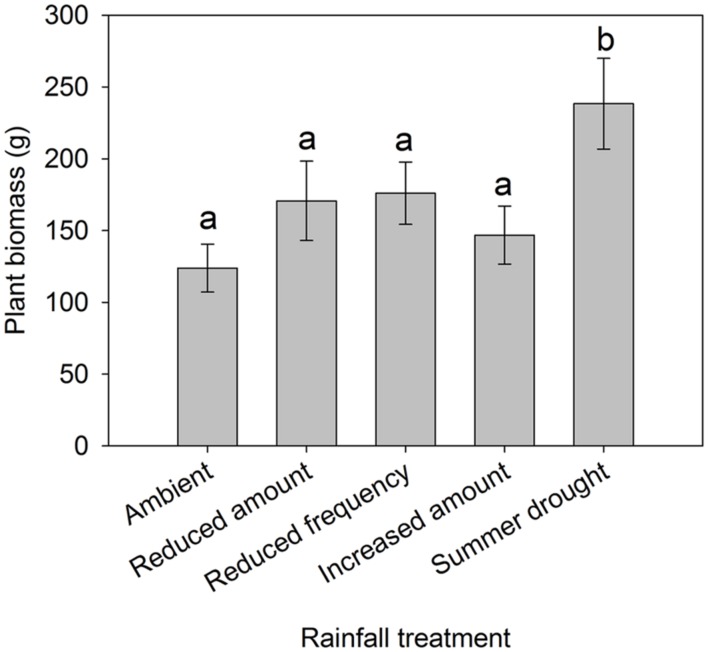
**Total aboveground plant biomass from grassland plots subjected to different rainfall regimes.** Bars with different letters (“a” and “b”) are significantly different from one another. Mean values ± SE shown (*N* = 12 for Ambient, Reduced amount, and reduced frequency, *N* = 6 for Increased amount and summer drought).

Irrigation regime significantly affected soil moisture which varied by month, as shown by the interaction (F_48,8700_ = 35.6, *P* < 0.001) (**Figure 1**). Plant biomass also varied between treatments ($\chi_{4}^{2}$ = 10.069, *P* = 0.039), with a greater plant biomass in the summer drought plots at the time of sampling, 5 months after the drought period ended (**Figure 2**). It was also in these plots that soil moisture decreased throughout the drought period, but returned to ambient levels in May, 1 month after the drought period ended (**Figure 1**; **Table 1**).

**Table 1:** Results from linear model *post hoc* test showing differences in soil moisture between ambient and summer drought plots.

Month	Estimate	Standard error	*Z* value	*P*-value
January (Summer drought started)	0.441	0.179	4.088	<0.001
February	0.589	0.108	5.442	<0.001
March (Summer drought terminated)	0.240	0.108	2.313	0.021
April	–1.365	0.1527	–8.937	<0.001
May	0.031	0.10787	0.291	0.771


*Soil moisture is shown for months during the summer drought and 2 months after the summer drought period.*

### Invertebrate Population Responses

**FIGURE 3:**
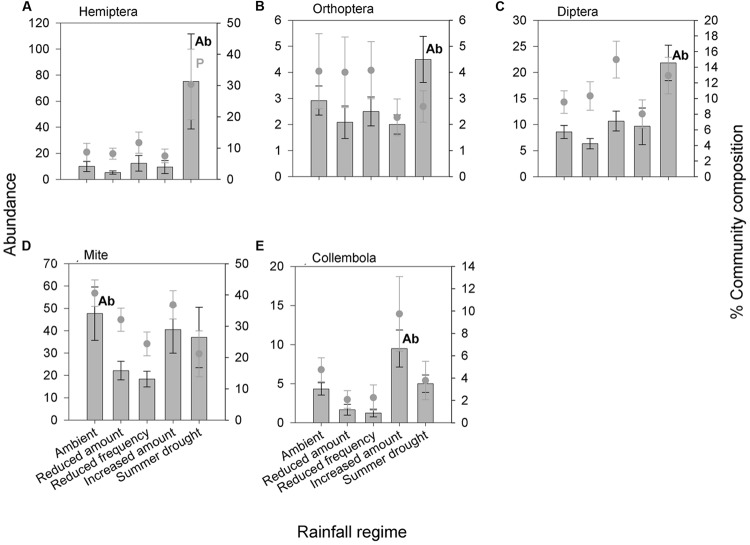
**Aboveground invertebrate taxonomic groups **(A)** Hemiptera, **(B)** Orthoptera, **(C)** Diptera, **(D)** Mite and **(E)** Collembola were significantly affected by rainfall regime in terms of absolute abundance and/or change in proportion of the community.** Bars show absolute abundance and points indicate the proportion of the overall community represented by the group. Statistically significant changes in absolute abundance indicated ‘Ab’ (see **Table 2** for corresponding analysis). Taxonomic groups most influencing the reported change in community composition indicated ‘P’. Mean values ± SE shown (*N* = 6).

**FIGURE 4:**
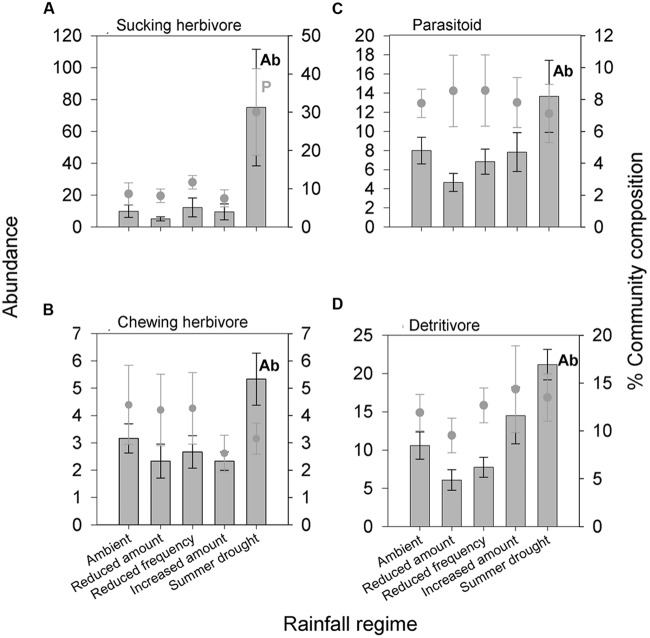
**Aboveground invertebrate feeding guilds **(A)** sucking herbivore, **(B)** parasitoid, **(C)** chewing herbivore, and **(D)** detritivores significantly affected by different rainfall regimes in terms of absolute abundance and/or change in proportion of the community.** Bars show absolute abundance and points indicate the proportion of the overall community represented by the group. Statistically significant changes in absolute abundance indicated ‘Ab’ (see **Table 2** for corresponding analysis). Feeding guilds most influencing the reported change in community composition indicated ‘P’. Mean values ± SE shown (*N* = 6).

In total, 6,604 aboveground invertebrates and 3,736 belowground macro invertebrates were counted and identified. A number of aboveground invertebrate taxa varied significantly in abundance between rainfall treatments (**Table 2**). Specifically, summer drought increased the abundance of invertebrates in the Orders Hemiptera, Orthoptera, Diptera, and Acari (**Table 2**; **Figures 3A–D**). In terms of functional guilds, summer drought positively affected the abundance of sucking herbivores, parasitoids, chewing herbivores, and detritivores (**Figures 4A–D**). The abundance of Collembola also varied between rainfall regimes, with a greater number found under the increased amount treatment (**Table 2**; **Figure 3E**). A number of taxonomic groups and guilds did not vary in abundance significantly between treatments (**Table 2**). The community composition of aboveground invertebrates varied significantly between rainfall treatments (**Table 3**). Despite a number of groups varying in their abundance, sucking herbivores and their associated order Hemiptera largely accounted for the change in community composition because of their very large increase in abundance under summer drought (**Figures 3** and **4**).

**Table 2:** Results from general linear model, showing how absolute abundance varied between rainfall treatments.

Community	Classification level	Figure reference	Community/Group	Likelihood ratio	*P*-value
Aboveground invertebrate macro fauna	Taxonomic classification		Community	16.466	**0.002**
			
		**Figure 3A**	Hemiptera	18.521	**<0.001**
		**Figure 3B**	Orthoptera (Poisson)	-10.510	**0.032**
		**Figure 3C**	Diptera	14.313	**0.006**
		**Figure 3D**	Collembola^∗^	18.979	**<0.001**
		**Figure 3E**	Acari^∗^	12.155	**0.016**
			Araneae	2.860	0.581
			Hymenoptera	6.806	0.147
			Coleoptera	1.564	0.815
			
	Feeding guild		Community	16.464	**0.002**
			
		**Figure 4A**	Sucking herbivore	18.094	**0.001**
		**Figure 4B**	Parasatoid	14.131	**0.006**
		**Figure 4C**	Chewing herbivore (Poisson)	-12.637	**0.013**
		**Figure 4D**	Detritivore	18.405	**0.001**
			Predator	1.694	0.791
			Scavenger	9.222	0.055
			Omnivore	2.367	0.669
			
Belowground invertebrate	Taxonomic classification		Community	5.991	0.199
			
		**Figure 5A**	Hymenoptera	12.242	**0.015**
			Diptera	6.438	0.168
			Hemiptera (Poisson)	-2.444	0.654
			Coleoptera	5.973	0.201
			Araneae	6.275	0.179
			Megadrilacea	3.845	0.427
			
	Feeding guild		Community	5.855	0.210
			
		**Figure 5B**	Scavenger	12.519	**0.014**
			Chewing herbivore	9.071	0.059
			Detritivore	3.647	0.455
			Omnivore	3.496	0.478
			Sucking herbivore	6.774	0.133
			Predator	5.044	0.28
			
Nematodes	Feeding guild		Community	3.462	0.177
			
			Fungal feeders	3.829	0.147
			Bacterial feeders	2.300	0.317
			Omnivore	0.734	0.693
			Plant parasites	3.774	0.152


*All groups have been classified to an order level with the exception of these groups marked with ^∗^ which have been classified to subclass. Statistically significant differences indicated in bold.*

**Table 3:** Results from multivariate permutational analysis (PERMANOVA) showing the effect of rainfall treatment on the community composition of above- and below-ground invertebrates at both order and guild level.

Group	Classification	d.f	Sum of squares	Mean of squares	Psuedo-F	*P*-value
Aboveground invertebrates	Order-level	4	0.885	0.221	2.265	**0.005**
	Guild- level	4	0.765	0.191	2.117	**0.016**
Belowground macro- invertebrates	Order-level	4	0.722	0.181	1.191	0.255
	Guild- level	4	0.718	0.179	1.205	0.268
Belowground nematodes	Order-level					
	Guild- level	2	0.060	0.030	0.397	0.892


*Belowground macro invertebrates and nematodes were analyzed separately due to differences in sampling techniques.*

**FIGURE 5:**
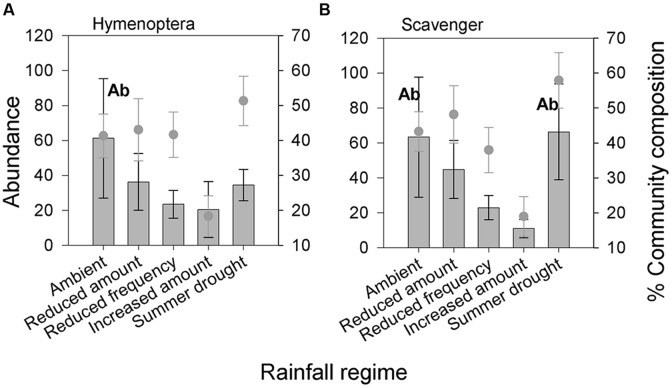
**Belowground invertebrate taxonomic groups and feeding guilds **(A)** Hymenoptera and **(B)** Scavengers significantly affected by different rainfall regimes in terms of absolute abundance.** Bars show absolute abundance and points indicate the proportion of the overall community represented by the group. Statistically significant changes in absolute abundance indicated ‘Ab’ (see **Table 2** for corresponding group. Mean values ± SE shown (*N* = 6).

Belowground Hymenoptera (Formicidae) and their associated guild, scavengers, were the only belowground groups for which abundance was significantly affected by altered rainfall regimes (**Table 2**, **Figures 5A,B**). The community composition of belowground fauna was not detectably affected by the rainfall treatments (**Table 3**).

### Exploring Above- and Below-Ground Linkages

**FIGURE 6:**
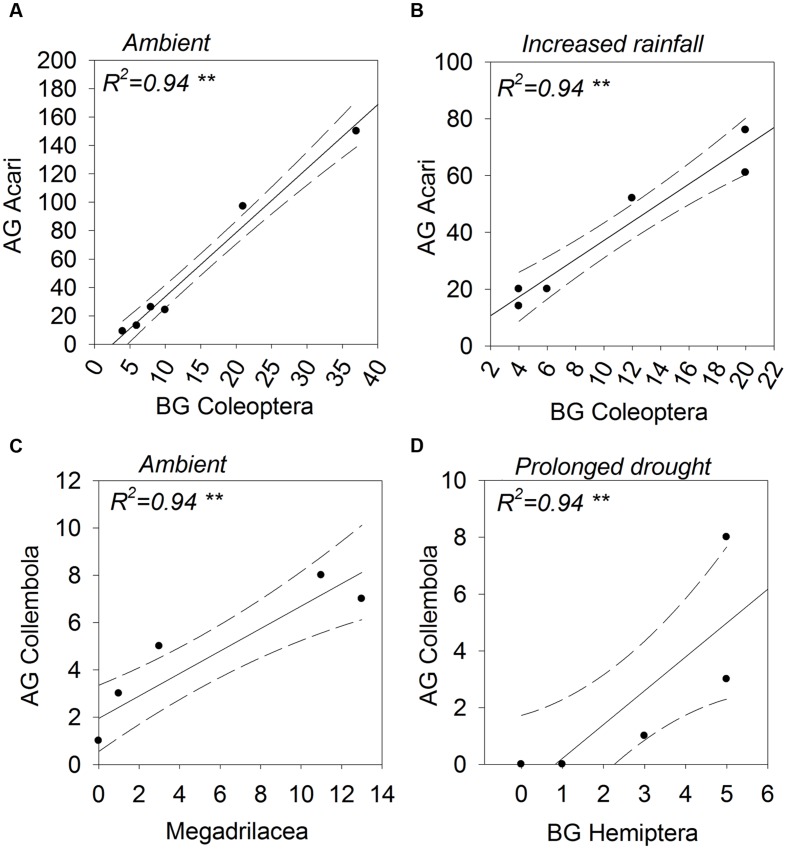
**Above- (shown on the *y*-axis) and below-ground (shown on the *x*-axis) taxonomic groups showing statistically significant correlations in abundance; **(A)** Acari (aboveground) and Coleoptera (belowground), **(B)** Acari (aboveground) and Coleoptera (belowground), **(C)** Collembola (aboveground) and Megadrilacea (belowground), and **(D)** Collembola (aboveground) and Hemiptera (belowground).** The rainfall regime under which the correlation occurred indicated for each panel. Solid lines indicate model predicted values; points represent actual values and dashed lines represent 95% confidence intervals.

**FIGURE 7:**
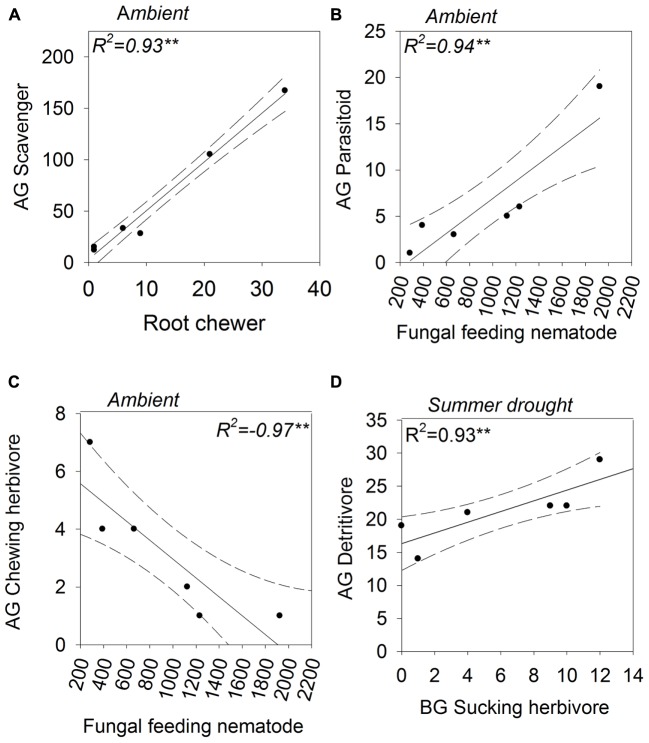
**Above- (shown on the *y*-axis) and below-ground (shown on the *x*-axis) feeding guilds showing statistically significant correlations in abundance; **(A)** scavengers (aboveground) and root chewers (belowground), **(B)** parasitoids (aboveground) and fungal feeding nematodes (belowground), **(C)** chewers (aboveground) and fungal feeding nematodes (belowground), and **(D)** detritivores (aboveground) and suckers (belowground).** The rainfall regime under which the correlation occurred indicated for each panel. Solid lines indicate model predicted values; points represent actual values and dashed lines represent 95% confidence intervals.

The abundances of aboveground Acari and belowground Coleoptera were positively correlated under ambient and increased amount rainfall scenarios (**Figures 6A,B**). The same relationship was observed for their associated guilds, scavengers and root chewers, under ambient rainfall regimes only (**Figure 7A**). A number of other relationships were only observed under ambient rainfall, including positive correlations between aboveground Collembola and belowground Megadrilacea (**Figure 6C**), and aboveground parasitoids and belowground fungal feeding nematodes (**Figure 7B**). Conversely, a negative relationship was found between the abundance of belowground fungal feeding nematodes and root chewers (**Figure 7C**). Additionally, the abundances of aboveground Collembola and belowground Hemiptera were positively correlated under the reduced amount treatment (**Figure 6D**), while a positive relationship was found between the abundance of aboveground detritivores and belowground sucking herbivores under summer drought conditions (**Figure 7D**). The abundance of a number of other aboveground and belowground invertebrates where found to have a strong correlation, however they did not appear to have a linear relationship (see **Supplementary Table 3**).

## Discussion

To our knowledge, this is the first report of a community-level field investigation of above- and below-ground invertebrate abundances across a range of potential future rainfall scenarios. In addition to reporting changes in the abundances of some invertebrates under altered rainfall, we showed that some above- and below-ground populations were tightly correlated, suggesting strong, yet precipitation-sensitive, linkages between these spatially separated organisms.

### Aboveground Invertebrate Responses

Of the four rainfall regimes, summer drought had the greatest effect on aboveground invertebrates, increasing the abundance of a number of invertebrate groups at both a taxonomic and guild level. These plots also had the greatest plant biomass, which is indicative of vigorous plant growth in the 5 months since the end of the summer drought. It is probable that there was more dieback in the 13 weeks without water during the growing season (January–March 2015). When precipitation returned to ambient levels, it is likely that resilient plants could take advantage of reduced competition and display vigorous growth. We propose that these results support the plant vigor hypothesis (Price, 1991). In particular, sucking herbivores (and Hemiptera) frequently show population spikes in spring because amino acids are being translocated for new growth (Dixon, 1998), so the more vigorous plant growth observed in summer drought plots benefited this group most.

Our observations of increased abundances of sucking herbivores in plots experiencing summer drought also has compatibilities with the pulsed-stress hypothesis (Huberty and Denno, 2004). Their meta-analysis demonstrated that sucking herbivores often benefit from plants subjected to intermittent water stress. In particular, reduced soil moisture often increases plant foliar nitrogen because pre-existing proteins can be hydrolysed, resulting in higher concentrations of free amino acids (Brodbeck and Strong, 1987). When rainfall resumes, phloem turgor pressure increases, allowing sucking herbivores (reliant on positive turgor pressure in the plant) to utilize the plant’s improved nutritional quality (Huberty and Denno, 2004). However, 5 months had elapsed since irrigation was applied at ambient levels, so we consider that increases in foliar nitrogen wouldn’t persist for so long. While it is possible that an increase in sucking herbivore abundance due to pulsed-drought in April underpinned larger populations observed in September, we find the plant vigor hypothesis more convincing, particularly since we also saw increases in other groups (e.g., chewing herbivores and detritivores). Moreover, our reduced frequency treatment (intermittent periods of drought, followed by larger rainfall events) is probably more similar to the pulsed-drought described by Huberty and Denno (2004), and we saw little impact on sap-suckers and Hemiptera in these plots.

The abundance of parasitoids also increased under summer drought, suggesting that parasitoids are tracking their Hemipteran hosts’ population dynamics, as found in Zhu et al. (2014). Indeed, the abundance of parasitoids was positively correlated with sucking herbivores in summer drought plots, providing further evidence that these insects are tracking the abundance of their hosts.

The general increase in herbivores under summer drought associated with the higher levels of cool season plant growth may explain the increased abundance of detritivores as well. In particular, the greater quantity of insect cadavers, frass and plant detritus is likely to have increased resources for this group. Increases in fungus gnats (Diptera), rather than Collembola, underpinned the increase in detritivores. Instead, the abundance of Collembola was more sensitive to increases in rainfall as evidenced by their greater numbers under the increased amount treatment compared with reduced frequency and amount plots. The abundance of Acari was reduced within the reduced frequency plots, indicating that this group may be sensitive to reductions in water availability. Both Collembola and Acari could have been negatively affected directly by the low soil moisture in the reduced rainfall plots (Convey et al., 2002; Lindberg et al., 2002; A’Bear et al., 2014).

In addition to changes in absolute abundance, the community composition of invertebrates also varied between rainfall treatments. Despite a number of aboveground invertebrates increasing in absolute abundance under summer drought plots, the observed changes in community composition aboveground were driven primarily by increases in the relative abundance of sucking herbivores (Hemiptera).

### Below-Ground Invertebrate Responses

Altered rainfall regimes had little measurable effect on the belowground community, with no change in community composition found between treatments. Altered precipitation patterns may have affected belowground invertebrates less because they have a host of adaptations to mitigate changes in their microclimate, such as utilizing metabolic water, moving through the soil profile and constructing earthen chambers (Barnett and Johnson, 2013). Only Hymenoptera (Formicidae) and their associated scavenger guild were affected by altered rainfall, showing reduced abundance in plots receiving increased amounts of rainfall. This could be the result of negative effects of increased soil humidity on ants, as suggested by Seal and Tschinkel (2010).

### Above- and Below-Ground Linkages

A number of above- and below-ground linkages were observed under ambient rainfall, but dissipated under altered rainfall regimes. The reasons for such linkages occurring and their decoupling under altered rainfall patterns remain unknown, and we stress that explanations postulated below are highly speculative. They do, however, provide the basis for formulating hypotheses to direct future work.

The positive relationship between the abundance of Acari (aboveground) and Coleoptera (belowground), for example, was only observed under ambient and increased amount plots. Root chewing herbivores like Coleopteran larvae can induce plants to reallocate resources aboveground, moving resources away from the site of attack, termed ‘resource sequestration’ (Orians et al., 2011; van Dam and Heil, 2011). Improved nutritional quality of plant detritus aboveground could then benefit scavengers, like Acari. The breakdown of these relationships could be a result of changes in the composition of species within these groups under altered rainfall regimes, even if the groups’ abundance didn’t change. Similarly, changes in plant community traits under drought (e.g., greater root:shoot ratios, accelerated senescence and litter production, and shifts in nutrient stoichiometry [Dijkstra et al., 2012]) could influence the plant-mediated aboveground-belowground interactions.

Collembola (aboveground) and Megadrilacea (belowground) were positively correlated under only ambient rainfall. Both groups are detritivores therefore their abundances may simply reflect similar responses to variations in the detritus inputs in ambient plots, resulting in a correlation. Supplementing detrital material has, for example, been shown to increase detritivore abundance (Halaj and Wise, 2002). Altered rainfall may have negatively affected aboveground detritivores to a greater extent than belowground detritivores, decoupling the association between the two groups.

The negative relationship between fungal feeding nematodes (belowground) and chewing herbivores (aboveground) was also only present under ambient rainfall; this finding may possibly be mediated via mycorrhizal communities. Fungal feeding nematodes can indicate the presence of mycorrhizal fungi in the soil (Landesman et al., 2011). Mycorrhizal infection frequency increases plant resistance to herbivory aboveground (Fontana et al., 2009), thereby potentially reducing the abundance of generalist chewing herbivores (Gange et al., 2002). More information on treatment effects on mycorrhizal root colonization and community composition would be required to support this theory. Altered precipitation regimes didn’t result in the association between herbivores and fungal feeding nematodes. Variation in the composition of mycorrhiza species, as well as colonization rates, under different rainfall regimes could have altered their effect on plant chemistry and the aboveground invertebrate community.

## Conclusion

This study indicates that changes in precipitation, specifically changes in the seasonality of rainfall, are likely to cause alterations in the abundance and composition of aboveground, and to a lesser extent belowground, invertebrate communities. In particular, summer drought resulted in outbreaks of sucking herbivores, which probably underpinned the concurrent increase in the abundance of parasitoids. However, this study represents a ‘snapshot’ of impacts on invertebrate communities in spring only and patterns may be different during the growing season. It remains to be determined whether these changes persist over the longer term or whether communities return to a state of equilibrium. These findings, together with the precipitation-sensitive linkages outlined in this study, do, however, lend further empirical support to the idea that climate change will modify grassland invertebrate communities with potential cascading effects. Moreover, linkages between above- and below-ground communities may be modified by climate change (McKenzie et al., 2013), which we propose can be termed ‘climatic decoupling.’

## Author Contributions

SJ conceived the experimental design. MT, KB, UN, SP, and SF collected field data. MT and SF analyzed the data with help from KB and SJ. MT wrote the paper with the assistance of KB, SF, UN, SP, and SJ.

## Conflict of Interest Statement

The authors declare that the research was conducted in the absence of any commercial or financial relationships that could be construed as a potential conflict of interest.
