# Supplementary material for: Altered Precipitation Impacts on Above- and Below-Ground Grassland Invertebrates: Summer Drought Leads to Outbreaks in Spring
Source: Front Plant Sci. 2016 Oct 6;7:1468. doi: 10.3389/fpls.2016.01468 (PMC5052266; doi:10.3389/fpls.2016.01468)
Supplement: Supplementary file 1 [file Table_1.DOCX]

**Supplementary Material**

**Sup. Table 1.** Groups of aboveground invertebrates identified, all groups identified to at least order level with the exception of those marked with an asterix which are identified to Subclass.

| Group identified | Feeding guild |
| --- | --- |
| Diptera:  Sciaridae  Other | Detritivore |
|  | Omnivores |
| Coleoptera:  Coccinellidae  Staphylinidae  Other | Predator |
|  | Predator |
|  | Omnivore |
| Araneae | Predators |
| Acari * | Scavengers |
| Hemiptera:  Predatory | Sucking herbivores  Predator |
| Other | Sucking |
| Hymenoptera:  Apocrita | Parasatoid |
| Formicidae | Scavenger |
| Orthoptera | Chewing herbivore |
| Lepidoptera | Chewing herbivore |
| Collembola* | Detritivore |

**Sup. Table 2.** Groups of belowground macro fauna identified, all groups identified to at least order level.

| Groups identified | Feeding guild |
| --- | --- |
| Diptera |  |
| Inopus spp. ( e.g Stratiomyidae) | Sucking Herbivore |
| Tabanidea | Predator |
| Sciaridae | Detritivore |
| Therevidea | Predators |
| Diptera other | Omnivores |
| Coleoptera |  |
| Scarabaeidea | Chewing herbivore |
| Elateridae | Omnivores |
| Tenebrionidea | Scavenger |
| Curculionidae | Chewing herbivore |
| Staphylinidea | Predators |
| Dung beetle | Detritivores |
| Beetle other | Omnivores |
| Hymenoptera |  |
| Formicidae | Scavengers |
| Hemiptera | Sucking herbivore |
| Myriapoda | Omnivore |
| Megadrilacea | Detritivores |
| Araneae | Predators |
| Blattodea | Scavengers |
| Lepidoptera | Chewing herbivore |
| Psocoptera | Detritivore |
| Isopoda | Detritivore |
| Orthoptera | Chewing herbivore |
| Mantodea | Predators |

**Sup. Table 3.** Mixed model results of highly significant above-belowground correlations (p=<0.001). Mixed model results which are significant are in bold, indicating a linear relationship, while these relationships are shown visually in Fig. 5 and 6.

| Group | Rainfall treatment | Significant correlation | | Mixed model results | |
| --- | --- | --- | --- | --- | --- |
|  |  | Aboveground group | Belowground Group | F value | P value |
| Taxonomic classification | Ambient | Acari | Coleopteran | 197.988 | **<0.001** |
|  |  | Collembola | Megadrilacea | 22.677 | **0.008** |
|  | Reduced amount | Collembola | Hemiptera | 8.020 | **0.0472** |
|  | Increased amount | Acari | Coleopteran | 57.063 | **0.002** |
|  |  | Orthoptera | Megadrilacea | 1.12 | 0.349 |
| Feeding guild | Ambient | Parasitoid | Fungal feeding nematodes | 16.885 | **0.014** |
|  |  | Scavenger | Chewing herbivore | 191.955 | **<0.001** |
|  |  | Chewing herbivore | Fungal feeding nematode | 12.082 | **0.025** |
|  | Increased amount | Omnivore | Chewing herbivore | 0.311 | 0.606 |
|  | Summer drought | Chewing herbivore | Detritivore | 0.122 | 0.744 |
|  |  | Detritivore | Sucking herbivore | 9.108 | **0.039** |
|  |  | Scavenger | Detritivore | 7.287 | 0.0541 |
